# Correlations between serotonin impairments and clinical indices in multiple system atrophy

**DOI:** 10.1111/ene.16158

**Published:** 2023-12-12

**Authors:** Ryunosuke Nagao, Yasuaki Mizutani, Sayuri Shima, Akihiro Ueda, Mizuki Ito, Junichiro Yoshimoto, Hirohisa Watanabe

**Affiliations:** ^1^ Department of Neurology Fujita Health University School of Medicine Toyoake Aichi Japan; ^2^ Department of Biomedical Data Science Fujita Health University School of Medicine Toyoake Aichi Japan; ^3^ International Center for Brain Science Fujita Health University Toyoake Aichi Japan

**Keywords:** 5‐hydroxyindoleacetic acid, cerebrospinal fluid, disease severity, multiple system atrophy, serotonergic neurons

## Abstract

**Background and purpose:**

Multiple system atrophy (MSA) is a neurodegenerative disease with characteristic motor and autonomic symptoms. Impaired brain serotonergic innervation can be associated with various clinical indices of MSA; however, the relationship between clinical symptoms and cerebrospinal fluid (CSF) levels of 5‐hydroxyindole acetic acid (5‐HIAA), a main serotonin metabolite, has not been fully elucidated.

**Methods:**

To compare CSF 5‐HIAA levels between patients with MSA and healthy controls, we included 33 controls and 69 MSA patients with either predominant parkinsonian or cerebellar ataxia subtypes. CSF 5‐HIAA levels were measured using high‐performance liquid chromatography. Additionally, we investigated correlations between CSF 5‐HIAA and various clinical indices in 34 MSA patients.

**Results:**

CSF 5‐HIAA levels were significantly lower in MSA patients than in controls (*p* < 0.0001). Probable MSA patients had lower CSF 5‐HIAA levels than possible MSA patients (*p* < 0.001). In MSA patients, CSF 5‐HIAA levels were inversely correlated with scores in Parts 1, 2, and 4 of the Unified Multiple System Atrophy Rating Scale, and with systolic and diastolic blood pressure in Part 3. Structural equation modeling revealed significant paths between serotonin and clinical symptoms, and significance was highest for activities of daily living, walking, and body sway.

**Conclusions:**

Serotonin dysfunction, as assessed by CSF 5‐HIAA levels, may implicate greater MSA severity.

## INTRODUCTION

Multiple system atrophy (MSA) is a sporadic, progressive neurodegenerative disease with an average onset age of approximately 60 years. It encompasses varying degrees of parkinsonism, cerebellar ataxia, autonomic failure, and pyramidal tract signs throughout its clinical course [1]. Although older onset cases are considered rare, recent investigations have revealed cases with ages of onset surpassing 75 years [2]. Patients with MSA can be classified as either predominant parkinsonism (MSA‐P) or cerebellar ataxia (MSA‐C) based on their predominant motor manifestations. The hallmark pathological feature of MSA is glial cytoplasmic inclusions within oligodendroglia; these inclusions comprise aggregated α‐synuclein [3].

There is currently no cure for MSA, and disease duration is typically approximately 9 years [4]. MSA is associated with a high incidence of sudden death [5], but a subset of patients have prolonged survival [6]. A meta‐analysis revealed that pronounced autonomic failure and the early onset of combined autonomic and motor manifestations are unfavorable prognostic indicators [7].

Although the precise pathogenesis underlying sudden death remains elusive, patients who undergo sudden death have more serotonergic neuronal loss within the brainstem [8, 9]. Furthermore, we have reported that serotonergic neuronal loss is prominent in autopsy cases with severe autonomic failure and sudden death but without distinct motor involvement [10]. Serotonin is involved in the control of not only respiratory, cardiovascular [11], and bladder function [12], but also gait function [13]; it may thus be related to the clinical features associated with MSA progression and prognosis.

The main metabolite of serotonin is 5‐hydroxyindole acetic acid (5‐HIAA). Although decreased cerebrospinal fluid (CSF) 5‐HIAA levels have been noted in MSA [14, 15, 16], the relationship between CSF 5‐HIAA levels and clinical features remains unclear. We therefore aimed to compare CSF 5‐HIAA levels between patients with MSA and controls, and to elucidate the correlations between CSF 5‐HIAA levels and diverse clinical indices in MSA.

## METHODS

### Participants

**FIGURE 1:**
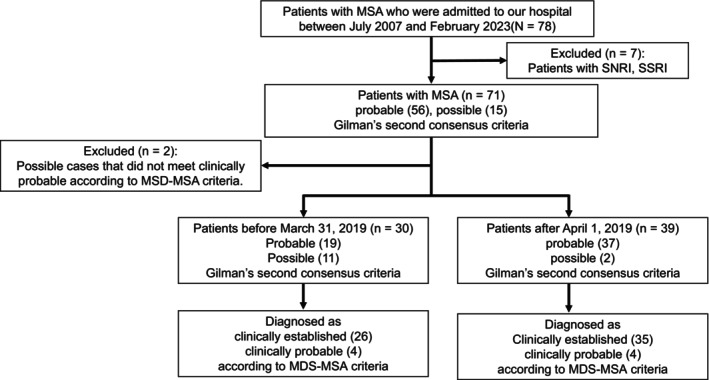
Study flow diagram. MDA, Movement Disorder Society; MSA, multiple system atrophy; SNRI, serotonin–norepinephrine reuptake inhibitor; SSRI, selective serotonin reuptake inhibitor.

We enrolled 69 patients diagnosed with MSA who were admitted to our hospital between July 2007 and February 2023. MSA was diagnosed according to Gilman's second consensus criteria [17]. Before 31 March 2019, we enrolled 30 cases that were not consecutive but had a diagnosis of probable MSA (19 cases) or possible MSA (11 cases) at the time of specimen collection. During subsequent follow‐up, seven of the possible MSA cases were diagnosed with probable MSA; the remaining four cases were still classified as possible MSA but were diagnosed with clinically probable MSA according to the Movement Disorder Society criteria for the diagnosis of MSA (MDS‐MSA criteria) published in 2022 [1]. From 1 April 2019, there were 39 consecutive cases, two of which were classified as possible MSA but were diagnosed as clinically probable based on the MDS‐MSA criteria. Currently, these patients are still under follow‐up. All 69 patients met the criteria for clinically probable or clinically established MSA according to the MDS‐MSA criteria (Figure 1).

Patients who received treatment with selective serotonin reuptake inhibitors (SSRIs) or serotonin–norepinephrine reuptake inhibitors were excluded. Of the 69 patients, 13 had possible MSA, 56 had probable MSA, 39 had MSA‐C, and 30 had MSA‐P. MSA‐P and MSA‐C were distinguished mainly based on the dominant symptoms observed during the examination, that is, whether they were related to parkinsonism or cerebellar ataxia. The mixed phenotype is diagnosed based on the primary symptoms, as evaluated by the physician in charge of the examination. There were 35 males and 34 females, with a mean age of 62.46 ± 8.17 years and a mean disease duration of 29.18 ± 18.72 months. Levodopa or dopamine agonist was administered to 31 patients, but none of them received monoamine oxidase type B inhibitor treatment. The total levodopa equivalent daily dose was calculated for each participant according to the established formula [18] (Table 1).

**TABLE 1:** Clinical characteristics of the participants.

Characteristic	Comparison study						Correlation study		
	Control	MSA	*p*	MSA‐P	MSA‐C	*p*	MSA	MSA‐P	MSA‐C
*n*	33	69		30	39		34	17	17
Male/female	22/11	35/34	0.1422^a^	14/16	21/18	0.2700^b^	17/17	8/9	9/8
Age at examination, years	63.27 ± 0.75	62.46 ± 8.17	0.4521^c^	62.43 ± 8.15	63.49 ± 8.29	0.7536^d^	62.18 ± 9.21	62.00 ± 9.30	62.53 ± 9.13
Probable/possible	NA	56/13	NA	28/2	28/11	**0.0304** ^e^	33/1	17/0	16/1
Age at onset, years	NA	59.84 ± 8.27	NA	59.50 ± 8.50	60.10 ± 8.19	0.7344^f^	58.90 ± 9.31	58.59 ± 9.56	59.65 ± 8.86
Disease duration, months	NA	29.18 ± 18.72	NA	28.07 ± 11.57	30.00 ± 22.75	0.4164^g^	31.67 ± 21.06	26.63 ± 13.00	36.41 ± 26.06
Dopaminergic treatment	NA	31	NA	23	8	**<0.0001** ^b^	21	15	6
LEDD, mg	NA	343.55 ± 160.07	NA	386.96 ± 30.01	218.75 ± 50.89	**0.0027** ^g^	221.21 ± 213.6	338.24 ± 211.79	96.88 ±132.25

*Note:* Bold indicates statistical significance.

Abbreviations: LEDD, levodopa equivalent daily dose; MSA, multiple system atrophy; MSA‐C, MSA‐cerebellar type; MSA‐P, MSA‐parkinsonian type; NA, not applicable.

^a^ Fisher exact test.

^b^ Comparison between the control group and the MSA‐P group and the MSA‐C group using Fisher's exact test.

^c^ The Wilcoxon rank sum test.

^d^ Comparison between the control group and the MSA‐P group and the MSA‐C group using the Kruskal–Wallis test.

^e^ Comparison between the MSA‐P group and the MSA‐C group using Fisher exact test.

^f^ Comparison between the MSA‐P group and the MSA‐C group using *t*‐test.

^g^ Comparison between the MSA‐P group and the MSA‐C group using the Wilcoxon rank‐sum test.

Additionally, we included 33 age‐matched controls (22 male and 11 female, mean age = 63.27 ± 9.75 years) who had no neurodegenerative disease and underwent epidural anesthesia for surgical treatment of urinary tract stones or prostatic hypertrophy in the urology department (Table 1).

### Measurement of CSF 5‐HIAA levels

Lumbar puncture was performed in all participants, and we measured CSF 5‐HIAA by high‐performance liquid chromatography method according to previously described methods [19]. For control studies, CSF specimens were centrifuged at 1500 × *g* for 10 min at 4°C within 2 h of collection, divided into 500‐μL portions, and then stored at −80°C. For MSA specimens, CSF was stored at 4°C after collection and measured within 1 day. The CSF 5‐HIAA level was considered unchanged based on previous literature on storage [20]. CSF 5‐HIAA was measured using high‐performance liquid chromatography with 1.5 mL of CSF [19]. The molecular weight of 5‐HIAA was calculated as 191 g/mol, and the measurement units were standardized. We compared CSF 5‐HIAA levels between 69 patients with MSA and 33 age‐matched controls in this comparative study.

### Clinical assessments

We next evaluated the correlations between CSF 5‐HIAA levels and diverse clinical indices in 34 of the 69 MSA patients (33 probable MSA and 1 possible MSA, 17 MSA‐C and 17 MSA‐P, 17 males and 17 females, mean age at onset = 62.18 ± 9.21 years, mean disease duration = 31.67 ± 21.06 months) who were admitted to our hospital between August 2020 and February 2023 (Table 2). In these patients, clinical MSA severity was assessed using the Unified Multiple System Atrophy Rating Scale (UMSARS). We also evaluated changes in systolic blood pressure, diastolic blood pressure, heart rate, and heart rate/systolic blood pressure in Part 3 of the UMSARS. Cognitive performance was evaluated using Addenbrooke's Cognitive Examination‐Revised, Frontal Assessment Battery, Mini‐Mental State Examination, and the Japanese version of the Montreal Cognitive Assessment. In addition to the UMSARS, motor and nonmotor symptoms were assessed using the Geriatric Depression Scale‐15, Odor Stick Identification Test for Japanese, and Scales for Outcomes in Parkinson's Disease‐Autonomic.

**TABLE 2:** Spearman correlation coefficient was utilized to examine the associations between CSF 5‐HIAA and clinical indices, with the exception of onset age, ΔsBP, ΔdBP, ΔHR, UMSARS Part 2, SCOPA‐AUT, and GDS, which were analyzed using Pearson correlation coefficient.

Clinical parameters	Mean ± SD	*r*s or *r*	*p*
CSF 5‐HIAA, ng/mL	9.87 ± 4.87		
Onset age, years	58.90 ± 9.31	0.2990	0.0859
Age at examination, years	62.18 ± 9.21	0.2118	0.2291
Disease duration, months	31.67 ± 21.06	0.1382	0.4358
**UMSARS Part I**	**17.28 ± 10.86**	**−0.5363**	**0.0019**
**UMSARS Part II**	**22.18 ± 9.35**	**−0.4447**	**0.0122**
**UMSARS Part III ΔsBP, mmHg**	**33.94 ± 17.72**	**−0.3537**	**0.0401**
**UMSARS Part III ΔdBP, mmHg**	**18.45 ± 12.26**	**−0.3860**	**0.0241**
UMSARS Part III ΔHR, bpm	7.71 ± 6.65	0.0874	0.6233
ΔHR/ΔsBP	0.232 ± 0.435	0.2526	0.1494
**UMSARS Part IV**	**2.43 ± 1.31**	**−0.4409**	**0.0102**
MMSE	26.62 ± 3.71	0.0364	0.8378
FAB	14.30 ± 2.54	0.1578	0.3727
MoCA‐J	22.18 ± 4.59	0.1691	0.3610
ACE‐R	86.06 ± 13.46	0.1263	0.4767
SCOPA‐AUT	15.68 ± 6.06	−0.20027	0.2561
GDS	8.15 ± 4.09	−0.22154	0.2080
OSIT‐J	9.43 ± 2.50	0.0805	0.6507

*Note:* Bold indicates statistical significance.

Abbreviations: 5‐HIAA, 5‐hydroxyindoleacetic acid; ACE‐R, Addenbrooke's Cognitive Examination‐Revised; CSF, cerebrospinal fluid; dBP, diastolic blood pressure; FAB, Frontal Assessment Battery; GDS, Geriatric Depression Scale‐15; HR, heart rate; MMSE, Mini‐Mental State Examination; MoCA‐J, Japanese version of the Montreal Cognitive Assessment; OSIT‐J, Odor Stick Identification Test for Japanese; sBP, systolic blood pressure; SCOPA‐AUT, Scales for Outcomes in Parkinson's Disease‐Autonomic; UMSARS, Unified Multiple System Atrophy Rating Scale.

### Ethical considerations

This study was approved by the ethics committee of Fujita Health University Hospital (HM22‐406). Controls were recruited in another study with ethical approval from the same committee (HG21‐015). Written informed consent, including the provision for opt‐out, was obtained from all participants prior to their inclusion. The study conforms to the World Medical Association Declaration of Helsinki.

### Statistical analysis

Statistical analyses were performed using JMP software (version 16, SAS Institute). Continuous variables are expressed as the mean ± SD. Differences were considered significant at *p* < 0.05. Fisher exact test was used to compare sex distributions and diagnostic classifications. The normality of variables was validated using the Shapiro–Wilk test. The Wilcoxon rank‐sum test was used to compare continuous variables between two groups, whereas the Kruskal–Wallis test was used to compare continuous variables among three groups; post hoc Steel–Dwass multiple comparison tests were then conducted on parameters with significant differences. Correlations between continuous variables, including CSF 5‐HIAA levels and clinical indices, were assessed using Spearman rank correlation coefficient and Pearson correlation coefficient in 34 MSA patients. We applied a false discovery rate (FDR) correction for multiple comparisons using the Benjamini–Hochberg procedure [21]. We also performed path analysis of the possible relationships between serotonin and clinical symptoms estimated by structural equation modeling to explore the pathogenesis of CSF 5‐HIAA reduction. We also performed structural equation modeling (SEM), which is a multivariate statistical analysis technique to analyze postulated causal relationships between variables. Once the path diagram is determined, the significance of each causality path can be evaluated by confirmatory factor analysis and multiple regression analysis [22]. An advantage of this analysis is that we can estimate latent relationships between serotonin and symptom domains. Also, we can emphasize interparticipant variation of the symptom assessment, because the latent score of each symptom domain is regarded as a composite score of its members. In this study, we investigated the relationship between serotonin levels in the brain and latent factors corresponding to symptom domains, which give rise to measurable scores of items in UMSARS Parts I and II using SEM. The path analysis was conducted using the Python package “semopy” [23].

## RESULTS

### Subject demographics

The statistical data and clinical characteristics of the 69 patients diagnosed with MSA and 33 controls are presented in Table 1. There were no significant differences in sex or age at examination between the MSA and control groups. Similarly, there were no significant disparities in age at onset, disease duration, or age at examination between the MSA‐P and MSA‐C groups. Furthermore, there were no notable variations in sex, age at onset, disease duration, or age at examination between patients with possible and probable MSA. Of the 13 cases classified as possible MSA, 12 displayed at least one characteristic magnetic resonance imaging (MRI) finding associated with MSA (such as the hot cross bun sign, middle cerebellar hyperintensity, putaminal atrophy, or dorsolateral putaminal hyper‐/hypointensities).

### Comparison of CSF 5‐HIAA levels between MSA and control groups

**FIGURE 2:**
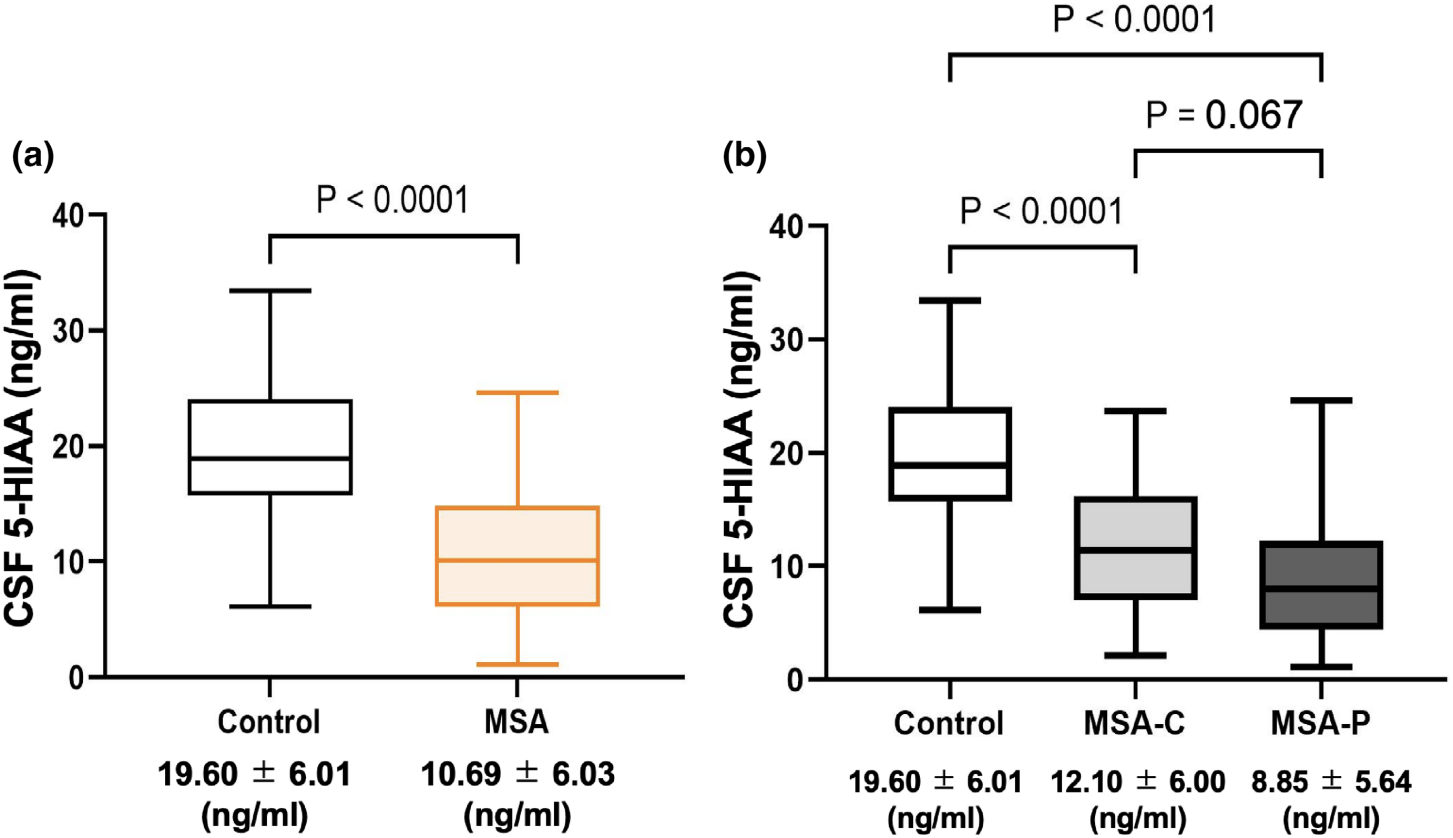
Comparison of cerebrospinal fluid (CSF) 5‐hydroxyindole acetic acid (5‐HIAA) levels between the control and multiple system atrophy (MSA) groups. (a) Comparison between the control and MSA groups using the Wilcoxon rank‐sum test. (b) Results of the Steel–Dwass test for multiple pairwise comparisons between the control group and the MSA‐C or MSA‐P group following the Kruskal–Wallis test. MSA‐C, MSA‐cerebellar type; MSA‐P, MSA‐parkinsonian type.

The CSF 5‐HIAA levels were significantly lower in the 69 MSA patients than in the controls (MSA: 10.69 ± 6.03 ng/mL; controls: 19.60 ± 6.01 ng/mL; *p* < 0.0001; Figure 2a). Both MSA‐P and MSA‐C patients had significantly lower CSF 5‐HIAA levels than the controls. Individuals with MSA‐P tended to have lower CSF 5‐HIAA levels than those with MSA‐C, although the difference was not significant (MSA‐P: 8.85 ± 5.64 ng/mL; MSA‐C: 12.10 ± 6.00 ng/mL; *p* = 0.067; Figure 2b). Moreover, CSF 5‐HIAA levels were markedly lower in patients with probable MSA than in those with possible MSA based on the second consensus statement (probable MSA: 9.31 ± 5.39 ng/mL; possible MSA: 16.60 ± 5.12 ng/mL; *p* < 0.001).

### Correlations between CSF 5‐HIAA levels and clinical MSA indices

Tables 2 and 3 show the clinical parameters of the 34 MSA patients, and the correlations between CSF 5‐HIAA and clinical indices. There were robust inverse relationships between CSF 5‐HIAA levels and the composite scores for Parts 1 (*r*s = −0.5363, *p* = 0.0019), 2 (*r* = −0.4447, *p* = 0.0122), and 4 (*r*s = −0.4409, *p* = 0.0102) of the UMSARS. Moreover, in Part 3, there were significant correlations between CSF 5‐HIAA levels and changes in systolic (*r* = −0.3537, *p* = 0.0401) and diastolic (*r* = −0.3860, *p* = 0.0241) blood pressure. There were no significant associations between CSF 5‐HIAA levels and general cognitive function as measured by the Mini‐Mental State Examination, Japanese version of the Montreal Cognitive Assessment, Addenbrooke's Cognitive Examination‐Revised, Frontal Assessment Battery, Geriatric Depression Scale‐15, or Odor Stick Identification Test for Japanese.

**TABLE 3:** Correlations between cerebrospinal fluid 5‐HIAA and clinical indices using Spearman rank correlation coefficient.

Clinical Index	Mean ± SD	*r*s	*p*	FDR (*q*‐value)
UMSARS Part 1				
1 Speech	1.39 ± 0.75	−0.32	0.066	0.122
2 **Swallowing**	**0.94 ± 0.86**	**−0.40**	**0.020**	**0.072**
3 Handwriting	1.33 ± 1.05	−0.29	0.100	0.145
4 Cutting food and handling utensils	1.24 ± 0.94	−0.34	0.051	0.113
5 **Dressing**	**1.42 ± 1.20**	**−0.41**	**0.019**	**0.072**
6 **Hygiene**	**1.52 ± 1.20**	**−0.44**	**0.010**	**0.072**
7 **Walking**	**2.06 ± 1.25**	**−0.43**	**0.013**	**0.072**
8 Falling	1.45 ± 1.48	−0.30	0.088	0.134
9 Orthostatic symptoms	1.52 ± 1.33	−0.34	0.056	0.113
10 **Urinary function**	**1.79 ± 1.32**	**−0.37**	**0.034**	**0.098**
11 Sexual function	1.76 ± 1.82	−0.34	0.054	0.113
12 Bowel function	1.64 ± 0.99	−0.28	0.121	0.158
UMSARS Part 2				
1 Facial expression	1.45 ± 1.00	−0.20	0.258	0.280
2 Speech	1.33 ± 0.74	−0.31	0.077	0.124
3 Ocular motor dysfunction	1.36 ± 0.82	−0.21	0.251	0.280
4 **Tremor at rest**	**0.52 ± 0.87**	**−0.42**	**0.015**	**0.072**
5 **Action tremor**	**1.18 ± 1.01**	**−0.40**	**0.022**	**0.072**
6 Increased tone	1.30 ± 0.95	−0.25	0.166	0.206
7 Rapid alternating movements of hands	1.79 ± 0.82	−0.28	0.111	0.152
8 Finger taps	1.82 ± 0.81	−0.32	0.073	0.124
9 Leg agility	1.73 ± 0.80	−0.23	0.198	0.234
10 Heel–knee–shin test	1.82 ± 0.85	−0.18	0.304	0.317
11 Arising from chair	1.55 ± 1.58	−0.34	0.057	0.113
12 Posture	1.42 ± 1.23	−0.17	0.348	0.348
13 **Body sway**	**2.06 ± 1.30**	**−0.41**	**0.016**	**0.072**
14 **Gait**	**2.24 ± 1.15**	**−0.41**	**0.018**	**0.072**

*Note:* Bold indicates statistical significance.

Abbreviations: FDR, false discovery rate; UMSARS, Unified Multiple System Atrophy Rating Scale.

**FIGURE 3:**
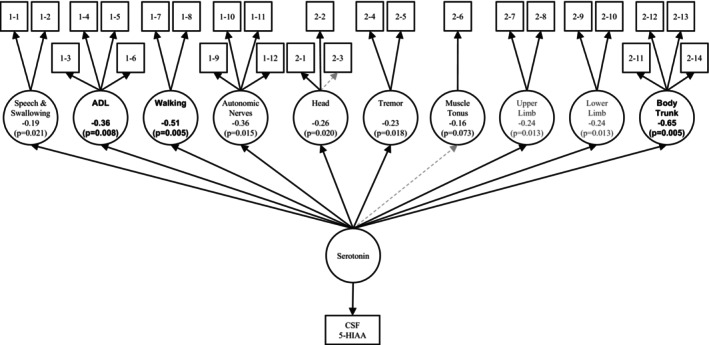
Path diagram of the possible relationships between serotonin and clinical symptoms estimated by structural equation modeling. The dashed gray lines are nonsignificant paths (*p* ≥ 0.05). The values associated with the circular nodes (latent factors) are regression coefficients with serotonin as a latent factor; those highlighted in bold are significant at *p* < 0.01. The numbers in the upper rectangles are the Unified Multiple System Atrophy Rating Scale item numbers: 1–1: speech; 1–2: swallowing; 1–3: handwriting; 1–4: cutting food and handling utensils; 1–5: dressing; 1–6: hygiene; 1–7: walking; 1–8: falling; 1–9: orthostatic symptoms; 1–10: urinary function; 1–11: sexual function; 1–12: bowel function; 2–1: facial expression; 2–2: speech; 2–3: ocular motor dysfunction; 2–4: tremor at rest; 2–5: action tremor; 2–6: increased tone; 2–7: rapid alternating movements of hands; 2–8: finger taps; 2–9: leg agility; 2–10: heel–knee–shin test; 2–11: arising from chair; 2–12: posture; 2–13: body sway; 2–14: gait. 5‐HIAA, 5‐hydroxyindoleacetic acid; ADL, activities of daily living; CSF, cerebrospinal fluid.

When analyzing the individual components of UMSARS Parts 1 and 2, there were significant correlations between CSF 5‐HIAA levels and swallowing, dressing, hygiene, walking, and urinary function in Part 1, as well as tremor at rest, action tremor body sway, and gait in Part 2. However, none of the items had an FDR below 0.05; they were all in the range of 0.05–0.1. The path diagram of the possible relationships between serotonin and clinical symptoms shows that there were many significant paths (*p* < 0.05). Especially, activities of daily living, walking, and body sway had high regression coefficients with serotonin as a latent factor (*p* < 0.01; Figure 3; Supporting Information).

## DISCUSSION

In the present study, there were significantly lower CSF 5‐HIAA levels in patients with MSA than in healthy controls. Furthermore, patients diagnosed with probable MSA had significantly lower CSF 5‐HIAA levels than those diagnosed with possible MSA. Our study also revealed inverse correlations between CSF 5‐HIAA levels and scores in Parts 1, 2, and 4 of the UMSARS, and between CSF 5‐HIAA levels and changes in blood pressure (UMSARS Part 3), suggesting that lower levels of CSF 5‐HIAA are associated with greater severity of MSA. Additionally, specific components of the UMSARS, including swallowing, dressing, hygiene, walking, urinary function, tremors, and gait, were significantly associated with CSF 5‐HIAA levels. However, none of the items had an FDR below 0.05; they were all in the range of 0.05–0.1. This indicates weak relationships between these subitems and 5‐HIAA. SEM revealed significant paths for a wide range of factors at the level of *p* < 0.05, and activities of daily living, walking, and body sway had regression coefficients with serotonin, as a latent factor, at the level of *p* < 0.01. Therefore, our results suggest that CSF 5‐HIAA levels may reflect the overall severity of MSA or the spread of lesions.

In the present study, the 69 patients with MSA had notably lower CSF 5‐HIAA levels than the controls. In previous studies, significant reductions in CSF 5‐HIAA levels were observed in 30 [14], 27 [15], and 16 [16] patients with MSA. Our findings, obtained in the largest sample size to date, thus confirm that CSF 5‐HIAA levels are decreased in MSA.

Our investigation also revealed a trend toward lower CSF 5‐HIAA levels in patients with MSA‐P than in patients with MSA‐C. The inclusion of “possible” MSA‐P cases in the present study (2/30 individuals; 6.7%), and of possible MSA‐C cases (11/39 individuals; 28.2%), may have affected these outcomes. Additionally, MSA‐C is more prevalent than MSA‐P in Japan. MRI can help diagnose MSA‐C even if autonomic failure is not severe. The higher prevalence of MSA‐C could be attributed to the abnormal findings thereof being easier to detect on MRI compared with those of MSA‐P [4]. Furthermore, a previous study of 19 MSA‐P and 26 MSA‐C patients did not identify any noteworthy distinctions between the two subtypes [24]. Nonetheless, our data indicate that patients with MSA exhibit serotonergic involvement regardless of clinical phenotype.

As shown in Table 2, we did not detect a correlation between disease duration and 5‐HIAA levels. This may be because each MSA patient progresses differently, and disease duration does not necessarily correlate with disease severity [25, 26]. Collecting multiple samples from the same patient would be helpful to investigate the correlation between CSF 5‐HIAA and disease duration. Unfortunately, we did not collect multiple samples from each patient.

### Autonomic symptoms and CSF 5‐HIAA levels in MSA

In the current study, lower CSF 5‐HIAA levels were related to more severe orthostatic hypotension (UMSARS Part 3) and urinary dysfunction (UMSARS Part 1). The effects of serotonin on blood pressure, as well as on the heart, kidney, and adrenal gland, are complex. Serotonin can cause vascular smooth muscle contraction, primarily via 5‐HT_1B_ and 5‐HT_2A_ receptors in smooth muscle cells. However, serotonin also has vasodilatory effects (mainly via 5‐HT_1B_ receptors) through the release of nitric oxide from endothelial cells [11]. Serotonin can either inhibit or stimulate preganglionic sympathetic nerves [27]. Furthermore, the effects of central nervous system neuronal serotonin deprivation on orthostatic hypotension have not been fully elucidated. In an animal model, long‐term fluoxetine treatment was effective against refractory neurogenic orthostatic hypotension [28]. However, a meta‐analysis revealed that SSRI use does not increase orthostatic hypotension frequency in the general population, irrespective of age [29]. Regarding the relationship between serotonin and urinary dysfunction, serotonin neurons in the medulla oblongata also regulate urinary reflexes [12]. Moreover, serotonin can induce bladder hypersensitivity via the activation of spinal 5‐HT_3_ receptors [30], and in a cohort study, lower serum serotonin levels were independently associated with an overactive bladder [31]. Thus, although there was no evidence of a direct relationship, our results indicate a possible relationship between serotonin and orthostatic hypotension and urinary dysfunction in MSA. However, additional clinicopathological studies are needed to clarify the causal role of serotonin in orthostatic hypotension and/or urinary dysfunction.

Benarroch et al. reported that patients with MSA who had severe autonomic failure, neurogenic bladder, and sleep apnea have a high degree of serotonin neuronal loss in the medulla oblongata [32]. Tada et al. observed significant loss of tryptophan hydroxylase immunoreactive neurons in the ventrolateral medulla and nucleus raphe obscurus among MSA patients who died suddenly without a clear cause compared with those whose cause of death was established. This result indicates that dysfunction of the medullary serotonergic system, which regulates the cardiovascular and respiratory systems, may be a potential cause of sudden death in patients with MSA [9].

Pathological studies of MSA patients with severe autonomic neuropathy and sudden death reported severe loss of tryptophan hydroxylase‐positive serotonergic neurons in the ventrolateral nucleus of the medulla and the nucleus raphe obscurus. Moreover, these patients had abundant glial cytoplasmic inclusions in the medullary reticular formations surrounding the nucleus raphe obscurus, the ventrolateral nucleus of the medulla, and the nucleus ambiguous. In classical MSA patients, these changes can range from benign to severe. These findings indicate that severe medullary serotonin neuron loss is associated with sudden death [10, 11].

Serotonin acts on chemical sensors that shape respiratory rhythms and are important for hypoxia and hypercapnia [33]. Prospective observational studies may therefore clarify the relationship between CSF serotonin levels and prognosis and/or sudden death in patients with MSA.

### Motor symptoms and CSF 5‐HIAA levels in MSA

In the present study, there were strong inverse associations between CSF 5‐HIAA levels and UMSARS scores for Parts 1, 2, and 4. Specifically, when we focused on each subitem of UMSARS Parts 1 and 2, there was a significant correlation between CSF 5‐HIAA levels and motor symptoms, such as body sway, gait abnormalities, tremor at rest, and action tremor, although none of the items had an FDR below 0.05; they were all in the range of 0.05–0.1, as stated above.

Descending neuromodulatory inputs from the brainstem can enhance the responsiveness of spinal motor neurons to inputs and prolong firing in the absence of synaptic inputs. To this end, motor neurons are densely innervated by serotonergic and noradrenergic inputs, and to a lesser extent by dopaminergic inputs [13]. For example, reduced serotonin levels in *Drosophila* affect the regulation of gait speed and environmental adaptation [34]. Moreover, dorsal raphe nucleus dysfunction may be associated with freezing of gait in Parkinson disease [35]. Serotonin dysfunction in the raphe nuclei in Parkinson disease is associated with the severity of resting tremor in the tremor‐predominant type [36], as well as with poor L‐dopa responsiveness [37] and action and postural tremors [38]. Although these previous studies focused on Parkinson disease rather than MSA, it is plausible that serotonin is also involved in the pathogenesis of tremors in MSA. Taken together, reduced CSF 5‐HIAA levels in MSA might reflect various motor manifestations and may serve as a reliable indicator of disease severity.

### Other clinical symptoms and CSF 5‐HIAA levels in MSA

In the current study, CSF 5‐HIAA levels were correlated with dysphagia severity. Similar to the respiratory and other autonomic systems, the role of serotonin in swallowing is complex because of the interplay between excitation and inhibition, and the involvement of several different serotonin receptors [39]. Furthermore, although mice with suppressed serotonin production in the brain develop dysphagia [40], the involvement of serotonin in dysphagia in MSA has been little investigated in humans or animal models.

In our study, there were no significant correlations between general cognitive performance and CSF 5‐HIAA levels. Although there is no clear link between serotonin and distinct behavioral and cognitive statuses, Shine et al. proposed that the central serotonergic system plays a crucial role in regulating cognitive processes by modulating brain activity [41]. Specifically, low serotonergic activity promotes cognitive automaticity, which allows for routine and habitual responses, whereas high serotonergic activity facilitates the generation of flexible solutions to problems, especially when the initial responses are ineffective. In Parkinson disease, it has been reported that serotonin transporter levels in the brain are inversely correlated with cortical amyloid‐β deposition, and that SSRI administration is associated with the prevention of cognitive decline [42]. The relationships between behavior, cognition, and serotonin may therefore need to be examined in MSA using a different battery than that of generalized cognitive function.

It has been reported that CSF 5‐HIAA levels are reduced in depressed patients [43]. However, there was no association between CSF 5‐HIAA levels and Geriatric Depression Scale‐15 scores in the present study, suggesting that lower CSF 5‐HIAA levels in MSA may be unrelated to mood.

### Limitations

This was a single‐center, cross‐sectional study. Furthermore, we did not investigate the relationship between CSF 5‐HIAA levels and sudden death or detailed respiratory function. In addition, we did not confirm our results using pathologically confirmed cases of MSA.

The results of this study suggest that the serotonin metabolite CSF 5‐HIAA is associated with scores on various clinical measures, indicating a potential link between the serotonergic system and the severity of MSA symptoms. Notably, however, these relationships may be nonspecific. To validate our findings, prospective studies correlating clinical imaging and pathological findings are needed using various methods, including measurement of CSF 5‐HIAA, serotonin and dopamine transporter imaging, quantitative MRI, and pathological studies.

### Prospects for the future

Although recent studies have reported that SSRIs may show some efficacy in MSA [44, 45], there is limited evidence regarding their effectiveness [46], and negative reports have also been published [47]. Nevertheless, the present findings suggest that interventions aimed at increasing brain serotonin levels may be valuable for treating MSA. Moreover, clinical trials using CSF 5‐HIAA levels as biomarkers are anticipated to enhance our understanding of the pathophysiology of MSA and facilitate the development of novel therapeutics. An exploration of noninvasive methods to visualize serotonin levels in the brain would be beneficial, considering the burdensome nature of spinal fluid testing.

## CONCLUSIONS

CSF 5‐HIAA levels were significantly lower in patients with MSA than in controls, and lower CSF 5‐HIAA levels were associated with more severe motor and autonomic symptoms. However, there were no significant correlations between cognitive function and mood. Together, these findings suggest that CSF 5‐HIAA levels may serve as a potential biomarker of disease severity in MSA.

## AUTHOR CONTRIBUTIONS

**Ryunosuke Nagao:** Writing – original draft; data curation; formal analysis; visualization; resources. **Yasuaki Mizutani:** Writing – review and editing; supervision; resources; formal analysis. **Sayuri Shima:** Resources. **Akihiro Ueda:** Resources. **Mizuki Ito:** Writing – review and editing; funding acquisition; supervision. **Junichiro Yoshimoto:** Software; formal analysis; data curation; methodology; validation. **Hirohisa Watanabe:** Writing – review and editing; supervision; conceptualization; funding acquisition; methodology; project administration.

## CONFLICT OF INTEREST STATEMENT

None of the authors has any conflict of interest to disclose.

## Supporting information


TABLE S1

## Data Availability

The data that support the findings of this study are available on request from the corresponding author. The data are not publicly available due to privacy or ethical restrictions.
