# Supplementary material for: Correlations between serotonin impairments and clinical indices in multiple system atrophy
Source: Eur J Neurol. 2023 Dec 12;31(3):e16158. doi: 10.1111/ene.16158 (PMC11235942; doi:10.1111/ene.16158)
Supplement: Supplementary file 1 — TABLE S1 [file ENE-31-e16158-s001.docx]

| lval | op | rval | Estimate | Std. Err | z-value | p-value | Bonf. p-value | FDR q-value |
| --- | --- | --- | --- | --- | --- | --- | --- | --- |
| Speech & Swallowing | ~ | serotonin | -0.188 | 0.082 | -2.301 | 0.021 | 0.214 | 0.024 |
| ADL | ~ | serotonin | -0.360 | 0.135 | -2.669 | 0.008 | 0.076 | 0.024 |
| Walking | ~ | serotonin | -0.514 | 0.182 | -2.823 | 0.005 | 0.048 | 0.024 |
| Autonomic Nerves | ~ | serotonin | -0.363 | 0.149 | -2.443 | 0.015 | 0.146 | 0.024 |
| Head | ~ | serotonin | -0.256 | 0.110 | -2.319 | 0.020 | 0.204 | 0.024 |
| Tremor | ~ | serotonin | -0.232 | 0.098 | -2.375 | 0.018 | 0.175 | 0.024 |
| Muscle Tonus | ~ | serotonin | -0.160 | 0.090 | -1.791 | 0.073 | 0.733 | 0.073 |
| Upper Limb | ~ | serotonin | -0.241 | 0.097 | -2.489 | 0.013 | 0.128 | 0.024 |
| Lower Limb | ~ | serotonin | -0.235 | 0.095 | -2.486 | 0.013 | 0.129 | 0.024 |
| Body Trunk | ~ | serotonin | -0.652 | 0.231 | -2.823 | 0.005 | 0.048 | 0.024 |
| UMSARS1_1 | ~ | Speech & Swallowing | 1.000 | - | - | - |  |  |
| UMSARS1_2 | ~ | Speech & Swallowing | 1.448 | 0.451 | 3.214 | 0.001 |  |  |
| UMSARS1_3 | ~ | ADL | 1.000 | - | - | - |  |  |
| UMSARS1_4 | ~ | ADL | 1.044 | 0.158 | 6.592 | 0.000 |  |  |
| UMSARS1_5 | ~ | ADL | 1.342 | 0.203 | 6.625 | 0.000 |  |  |
| UMSARS1_6 | ~ | ADL | 1.334 | 0.204 | 6.548 | 0.000 |  |  |
| UMSARS1_7 | ~ | Walking | 1.000 | - | - | - |  |  |
| UMSARS1_8 | ~ | Walking | 1.149 | 0.150 | 7.675 | 0.000 |  |  |
| UMSARS1_9 | ~ | Autonomic Nerves | 1.000 | - | - | - |  |  |
| UMSARS1_10 | ~ | Autonomic Nerves | 1.258 | 0.239 | 5.260 | 0.000 |  |  |
| UMSARS1_11 | ~ | Autonomic Nerves | 0.772 | 0.320 | 2.413 | 0.016 |  |  |
| UMSARS1_12 | ~ | Autonomic Nerves | 0.508 | 0.173 | 2.934 | 0.003 |  |  |
| UMSARS2_1 | ~ | Head | 1.000 | - | - | - |  |  |
| UMSARS2_2 | ~ | Head | 0.953 | 0.277 | 3.448 | 0.001 |  |  |
| UMSARS2_3 | ~ | Head | 0.099 | 0.249 | 0.396 | 0.692 |  |  |
| UMSARS2_4 | ~ | Tremor | 1.000 | - | - | - |  |  |
| UMSARS2_5 | ~ | Tremor | 1.472 | 0.363 | 4.059 | 0.000 |  |  |
| UMSARS2_6 | ~ | Muscle Tonus | 1.000 | - | - | - |  |  |
| UMSARS2_7 | ~ | Upper Limb | 1.000 | - | - | - |  |  |
| UMSARS2_8 | ~ | Upper Limb | 0.862 | 0.183 | 4.713 | 0.000 |  |  |
| UMSARS2_9 | ~ | Lower Limb | 1.000 | - | - | - |  |  |
| UMSARS2_10 | ~ | Lower Limb | 0.947 | 0.267 | 3.546 | 0.000 |  |  |
| UMSARS2_11 | ~ | Body Trunk | 1.000 | - | - | - |  |  |
| UMSARS2_12 | ~ | Body Trunk | 0.421 | 0.133 | 3.170 | 0.002 |  |  |
| UMSARS2_13 | ~ | Body Trunk | 0.827 | 0.090 | 9.190 | 0.000 |  |  |
| UMSARS2_14 | ~ | Body Trunk | 0.728 | 0.080 | 9.076 | 0.000 |  |  |
| CSF_5_HIAA | ~ | serotonin | 1.000 | - | - | - |  |  |

Supplemental information.

Plausible relationship between serotonin and clinical symptoms estimated by structural equation modeling.

Abbreviation: lval: left-side value; rval: right-side value; op: operator; ~:regression operator; Std. Err: standard error; Bonf.: Bonferroni; FDR: False Discovery Rate; UMSARS: unified MSA rating scale; CSF: cerecrospinal fluid; 5-HIAA: 5-hydroxyindoleacetic acid.
